# Hepatoprotective Effect of Polyphenol-Enriched Fraction from *Folium Microcos* on Oxidative Stress and Apoptosis in Acetaminophen-Induced Liver Injury in Mice

**DOI:** 10.1155/2017/3631565

**Published:** 2017-05-23

**Authors:** Hongtan Wu, Gang Zhang, Lisen Huang, Haiyue Pang, Na Zhang, Yupei Chen, Gueyhorng Wang

**Affiliations:** ^1^Application Technique Engineering Center of Natural Cosmeceuticals, College of Fuijan Province, Xiamen Medical College, Xiamen, Fujian 361023, China; ^2^Research Center of Natural Cosmeceuticals Engineering, Xiamen Medical College, Xiamen, Fujian 361023, China; ^3^Fujian Provincial Key Laboratory of Biological Engineering on Traditional Herbs, Xiamen Medical College, Xiamen, Fujian 361023, China; ^4^Technology and Engineering Center for Marine Biomedical Resource Utilization, Xiamen Medical College, Xiamen, Fujian 361023, China; ^5^Department of Pharmacy, Xiamen Medical College, Xiamen, Fujian 361023, China

## Abstract

*Folium Microcos* (FM), the leaves of *Microcos paniculata* L., shows various biological functions including antioxidant activity and *α*-glucosidase inhibitory effect. However, its therapeutic potential in acute liver injury is still unknown. This study investigated the hepatoprotective effect and underlying mechanisms of the polyphenol-enriched fraction (FMF) from *Folium Microcos*. FMF exhibited strong free radical scavenging activities and prevented HepG2/Hepa1–6 cells from hydrogen peroxide- (H_2_O_2_-) induced ROS production and apoptosis in vitro. Antioxidant activity and cytoprotective effects were further verified by alleviating APAP-induced hepatotoxicity in mice. Western blot analysis revealed that FMF pretreatment significantly abrogated APAP-mediated phosphorylation of MAPKs, activation of proapoptotic protein caspase-3/9 and Bax, and restored expression of antiapoptotic protein Bcl2. APAP-intoxicated mice pretreated with FMF showed increased nuclear accumulation of nuclear factor erythroid 2-related factor (Nrf2) and elevated hepatic expression of its target genes, NAD(P)H:quinine oxidoreductase 1 (NQO1) and hemeoxygenase-1(HO-1). HPLC analysis revealed the four predominantly phenolic compounds present in FMF: narcissin, isorhamnetin-3-O-*β*-D-glucoside, isovitexin, and vitexin. Consequently, these findings indicate that FMF possesses a hepatoprotective effect against APAP-induced hepatotoxicity mainly through dual modification of ROS/MAPKs/apoptosis axis and Nrf2-mediated antioxidant response, which may be attributed to the strong antioxidant activity of phenolic components.

## 1. Introduction

The liver is a major organ that regulates homeostasis of metabolism and detoxifies metabolites/xenobiotics generated during drug exposure in the body [1]. Owing to these crucial functions, the liver is liable to damages caused by hepatotoxic chemicals, free radicals, and other reactive oxygen species (ROS) [2]. Overproduction of pro-oxidants/ROS in the liver can cause damage to structural and functional integrity of cells, leading to extensive liver injury [3]. Dysfunction of antioxidant/detoxification defense system is also considered to play a significant pathophysiological role in the development of liver diseases [4]. Therefore, developing hepatoprotective agents to reduce or eliminate ROS production and to strengthen physiological antioxidant capability will be an effective strategy for fighting oxidative stress that causes liver damage and diseases.

Acetaminophen (N-acetyl-p-aminophenol, APAP), a commonly used analgesic and antipyretic drug, is widely used to investigate liver toxicity associated with oxidative stress and to evaluate therapeutic potential of drug candidates. APAP is usually safe and effective at therapeutic doses, but overdose of it can cause severe acute liver injury [5]. Mechanistically, APAP-induced hepatotoxicity is initiated by extensive formation of N-acetyl-p-benzoquinone imine (NAPQI), a reactive metabolite of APAP, which in turn depletes hepatic glutathione (GSH) and causes mitochondrial dysfunction and damage, resulting in overproduction of mitochondrial ROS [6, 7]. During APAP challenge, ROS generated from mitochondria and other sources not only causes cell damage via mechanisms involving lipid peroxidation with subsequent liver injury [8] but also activates many kinases, such as c-jun-N-terminal kinase (JNK), to aggravate degenerative progression in liver tissues [9]. Activated JNK binding to mitochondria further enhances ROS generation. In addition, JNK can phosphorylate several transcription factors, such as p53 and NF-*κ*B, to accelerate hepatic apoptosis and inflammatory response [10, 11], ultimately leading to extensive oxidative stress and acute liver injury.

Therefore, rigorous control of ROS levels by antioxidant molecules and detoxifying enzymes plays a crucial role in maintaining intracellular redox homeostasis in response to oxidative insults. It has been demonstrated that nuclear factor erythroid 2-related factor (Nrf2) and its negative regulator kelch-like ECH-associated protein 1 (Keap1) serve as an important reaction axis for cells to combat oxidative stress [12]. Nrf2 belongs to the basic leucine zipper family of transcription factors and is implicated as a major regulator of antioxidant response element- (ARE-) mediated detoxification and antioxidant gene expression [13]. Under basal conditions, Nrf2 interacts with Keap1 and is retained in the cytoplasm. In response to oxidative stress, Nrf2 dissociates from Keap1 and translocates to the nucleus, where it binds to ARE in the upstream region and subsequently promotes transcription of its target genes, including NAD(P)H:quinine oxidoreductase 1 (NQO1), glutamate-cysteine ligase catalytic and modifier subunits (GCLC and GCLM), and heme oxygenase-1 (HO-1) [14]. Considerable research has demonstrated the involvement of Keap1-Nrf2-ARE signaling pathway in organ injuries caused by toxic stimuli. Mice deficient in Nrf2 showed high susceptibility to APAP-induced liver injury [15, 16]. On the contrary, hepatocyte-specific Keap1 knockout mice exhibited an increase in genes encoding for detoxifying enzymes and resisted hepatotoxicity [17]. Thus, agents that can activate Nrf2 may be beneficial in mitigating oxidative stress-induced liver injury.

Despite the increasing need for hepatoprotective agents to protect people from liver injury, only a limited number of efficacious and reliable drugs were used successfully, and some of them even possessed potential adverse effects. In recent years, numerous edible and medicinal plants around the world have drawn increasing attention as dietary supplements and therapeutic intervention strategies for their excellent health-promoting properties and being less harmless than synthetic agents [18–20]. *Microcos paniculata* L., belonging to the Malvaceae family, is mainly distributed in tropical and subtropical areas of South and Southeast Asia [21]. In China, its leaves, named as *Folium Microcos* (FM), are traditionally used as folk medicine and herbal tea materials for treatment of fever, heatstroke, indigestion, and diarrhea [22]. Previous phytochemical studies on FM validated the existence of various bioactive constituents, such as triterpenoids, flavonoids, and alkaloids with pharmacological effects [21–23]. However, there are few reports available to describe the potential effect of FM against liver injury and what kinds of compounds it is related to. Hence, the present study evaluated the effect of the polyphenol-enriched fraction (FMF) from FM on oxidative stress and APAP-induced hepatotoxicity and investigated the potential mechanisms. Moreover, the main components of FMF were identified and quantified by reversed phase high-performance liquid chromatography (RP-HPLC) to gain insights into the compounds responsible for its antioxidant and hepatoprotective effects.

## 2. Materials and Methods

### 2.1. Chemicals and Reagents

Acetaminophen (N-acetyl-p-aminophenol, APAP), 3-(4,5-dimethylthiazol-2yl)-2,5-diphenyltetrazolium bromide (MTT), 2′,7′-dichlorodihydrofluorescein diacetate (DCFH-DA), 1,1-diphenyl-2-picrylhydrazyl (DPPH), nitroblue tetrazolium (NBT), phenazine methosulfate (PMS), *β*-nicotinamide adenine dinucleotide, reduced disodium salt, trihydrate (*β*-NADH), hematoxylin, and eosin were purchased from Sigma-Aldrich (St. Louis, MO, USA); Trizol, PrimeScript™ RT Master Mix, and SYBR Green Master Mix were purchased from Takara Biotechnology (Dalian, China); Oligonucleotides were synthesized by Sangon Biotech (Shanghai, China); Gallic acid and ascorbic acid were purchased from Sangon Biotech (Shanghai, China); assay kits of alanine aminotransferase (ALT), aspartate aminotransferase (AST), lactate dehydrogenase (LDH), catalase (CAT), superoxide dismutase (SOD), malonaldehyde (MDA), reduced glutathione (GSH), and glutathione peroxidase (GSH-Px) were purchased from Nanjing Jiancheng Institute of Biotechnology (Nanjing, China); Nuclear and Cytoplasmic Protein Extraction Kit was purchased from the Beyotime Institute of Biotechnology (Shanghai, China); Dulbecco's minimum essential medium (DMED), fetal bovine serum (FBS), and penicillin/streptomycin were purchased from Invitrogen (Carlsbad, CA, USA). All other chemicals were of the highest grade available.

### 2.2. Plant Material, Extraction, and Preparation

Leaves of *Microcos paniculata* L. were purchased from Guangzhou Qingping Professional Market for Traditional Chinese Medicine, Guangdong, China. The concentrated solution (FME) was prepared according to the following procedures [24]: the air-dried leaves of FM (400 g) were powdered and boiled with 7000 mL of deionized water for 1 h. The decoction was filtered and then concentrated by a rotary evaporator with a water bath at 55°C. FME was filtered again before being added to a Diaion HP-20 resin (Mitsubishi Chemical, Tokyo, Japan) column, which was then washed sequentially with two-column volumes of water and 75% aqueous ethanol. Sugars, proteins, and salts were removed by washing the column with water, and the eluate was named as FMW. Polyphenols were retained and then eluted with 75% aqueous ethanol, and the eluate was designated as FMF. Each eluate was concentrated by vacuum rotary evaporation. The concentrated solution was dried in a lyophilizer and then stored at 4°C. The yields of FMW and FMF were 20.0 and 10.9 g, respectively.

### 2.3. Determination of Total Polyphenols and Total Flavonoids

Total polyphenolic contents in the different fractions were measured by Folin-Ciocalteu method as gallic acid equivalents (GAE), expressed as milligrams of gallic acid per gram of fractions [25]. Briefly, aliquots of 1 mL samples or standard solutions were mixed with 1 mL of Folin-Ciocalteu reagent and allowed to react for 3 min. Then, 1 mL of 10% Na_2_CO_3_ solution was added to the mixture, and the distilled water was supplied to the final volume of 10 mL, followed by a thorough mixture and a further stand at 25°C for 2 h. Absorbance was detected at 760 nm, and total polyphenolic contents were calculated as GAE from a calibration curve (*y* = 0.0575*x* + 0.159, *R*^2^ = 0.9980, 1–50 *μ*g of gallic acid). Data were presented as the average of triplicate analyses.

Total flavonoids of the fractions were measured using a modified colorimetric method as rutin equivalents (RE), expressed as milligrams of rutin per gram of fractions [26]. Approximately 1 mL of samples or standard solutions were mixed with 0.2 mL of 5% NaNO_2_ solution. After 5 min, 0.2 mL of 10% AlCl_3_ solution was added, and the mixture was allowed to stand for another 5 min. Subsequently, the reaction solution was mixed with 0.6 mL of 4% NaOH solution, and 60% ethanol was immediately supplied to obtain a final volume of 10 mL, followed by thorough mixing and standing for another 10 min. Absorbance of the mixture was determined at 510 nm, and total flavonoid contents were calculated as RE according to a calibration curve (*y* = 0.0051*x* + 0.0029, *R*^2^ = 0.9999, 1–50 *μ*g of rutin). Data were presented as the average of triplicate analyses.

### 2.4. Measurement of Antioxidant Activities In Vitro

DPPH free radical scavenging activities of different fractions were evaluated based on a reported method [27]. One mL of samples at various concentrations was added to 1 mL of 100 *μ*M freshly prepared DPPH radical methanol solution. Reaction mixtures were shaken vigorously and incubated at 37°C in the dark for 30 min, and an equal volume of methanol and DPPH served as a control. Absorbance was measured at 517 nm. Superoxide anion radical (O_2_^•−^) scavenging activities of different fractions were determined using a modified method [28]. One mL of samples at various concentrations was successively mixed with 1 mL of 468 *μ*M NADH, 60 *μ*M PMS, and 156 *μ*M NBT and then incubated at room temperature for 5 min. Absorbance of the mixture was determined at 560 nm, and an equal volume of methanol served as a control.

Scavenging activities were estimated based on percentages of scavenged DPPH and O_2_^•−^, as shown by the following equation:

(1) $$\begin{array}{c} Scavenging\;effect\;\left(\%\right)=\frac{1-\left(absorbance\;of\;sample-absorbance\;of\;blank\right)}{\left(absorbance\;of\;control-absorbance\;of\;blank\right)}\times 100\%. \end{array}$$

### 2.5. Cell Culture and Treatment

Human hepatic carcinoma cell line (HepG2) and mouse hepatoma cell line (Hepa1–6) were purchased from American Type Culture Collection (ATCC, Manassas, VA, USA). Cells were cultured in DMEM supplemented with 10% FBS and 100 U/mL penicillin/streptomycin in humidified incubator at 37°C and 5% CO_2_-enriched atmosphere. To determine the antioxidant effect of FMF in H_2_O_2_-induced oxidative stress model, cells were seeded in 96-well plates at an initial density of 2 × 10^4^ cells/well to adhere overnight, incubated with various concentrations of FMF for 12 h, and then exposed to 400 *μ*M H_2_O_2_ without changing the medium for 4 h [29]. Control cells were incubated with culture medium of equal volume.

### 2.6. Cell Morphology Assay

Cells were seeded in 96-well plates at an initial density of 2 × 10^4^ cells/well to adhere overnight, incubated with various concentrations of FMF for 12 h, and then exposed to 400 *μ*M H_2_O_2_ without changing the medium for 4 h. Cell morphology images were taken at the magnification of 100x [29].

### 2.7. Cell Viability Assay

Cell viability was measured by MTT assay [30], which is based on the conversion of MTT to dark-blue formazan crystals by mitochondrial dehydrogenase enzyme. In brief, 10 *μ*L of 5 mg/mL MTT was added into each well and incubated at 37°C for 4 h. After incubation, the culture medium was removed from the wells and replaced with 150 *μ*L DMSO. Plates were then vigorously shaken to ensure complete solubilization. Finally, absorbance of formazan was measured on a plate reader (Molecular Devices, Sunnyvale, CA) at 490 nm. Relative cell viability was calculated as absorbance of sample-treated cells divided by absorbance of untreated control cells.

### 2.8. Measurement of Intracellular ROS In Vitro

Intracellular ROS levels were measured by using a modified method with DCFH-DA as a fluorescent probe [31]. Cells were cultured in collagen-coated 96-well black plates with transparent bottoms, treated with FMF at different concentrations for 12 h, and then exposed to 400 *μ*M H_2_O_2_ without changing the medium for 4 h. Cells were subsequently loaded with 10 *μ*M DCHF-DA at 37°C for 30 min. After washing twice with PBS buffer, fluorescence intensity that is representative of intracellular ROS levels was detected at 485/20 nm excitation and 528/20 nm emission using a fluorescence microplate reader (Molecular Devices, Sunnyvale, CA).

### 2.9. Animals and Experimental Design

Male ICR mice weighing 22.5 ± 1.0 g were purchased from the Laboratory Animal Center, Xiamen University (Xiamen, China). Mice were allowed to acclimate to laboratory conditions for 7 days prior to dosing and maintained in a temperature-controlled environment (22 ± 2°C) with a 12 h light-dark cycle, free access to water, and standard rodent chow. All methods were conducted in accordance with the National Institutes of Health Guidelines for the Care and Use of Laboratory Animals. All efforts were made to minimize the number of animals used and their suffering.

Mice were randomly divided into three groups and are as follows: Group 1, control group, given appropriate vehicle throughout the experiment; Group 2, APAP group, given 500 mg/kg body weight APAP; Group 3, APAP + FMF group, given different doses of FMF (100, 200, and 400 mg/kg body weight) prior to APAP administration.

Concretely, Groups 1 and 2 were given PBS, and Group 3 was orally administered with FMF once daily for 7 consecutive days. Two hours after the final administration, Group 1 was treated with appropriate vehicle, while Groups 2 and 3 were treated with APAP at a dose of 500 mg/kg body weight by intragastric administration. Mice were subsequently anesthetized for blood sample collection and then sacrificed to obtain liver tissues after 12 h APAP treatment. Serum samples were separated from the blood by centrifugation at 10,000 ×g for 10 min at 4°C and then kept at −80°C for bioassays. A portion of the liver was fixed by 4% paraformaldehyde for histopathological analysis, and the remaining tissues were flash-frozen in liquid nitrogen and stored at −80°C for further analysis.

### 2.10. Measurement of Biochemical Parameters in Serum

To assess liver injury caused by APAP administration, enzymatic activities of serum ALT, AST, and LDH were estimated by using the corresponding commercial kits (Nanjing Jiancheng Institute of Biotechnology, China). Results of ALT, AST, and LDH were expressed as units per liter (U/L).

### 2.11. Measurement of Hepatic Levels of GSH, GSH-Px, SOD, CAT, and MDA

Liver tissues were homogenized in ice-cold PBS buffer and then centrifuged at 10,000 ×g for 10 min at 4°C. Supernatants were collected for the measurement of hepatic MDA, GSH, GSH-Px, SOD, and CAT using the corresponding commercial kits (Nanjing Jiancheng Institute of Biotechnology, China). Results were corrected for their protein content.

### 2.12. Histopathological Analysis

Portions of the freshly obtained liver were fixed in 4% buffered paraformaldehyde phosphate solution for 24 h and then embedded in paraffin for sectioning [8]. Hematoxylin and eosin staining was performed on 5 *μ*m paraffin sections according to a standard procedure and analyzed by light microscopy.

### 2.13. Preparation of Nuclear and Cytosol Fractions

Liver nuclear and cytosol extracts were prepared by using the Nuclear and Cytoplasmic Protein Extraction Kit (Beyotime Institute of Biotechnology, Shanghai, China) according to the manufacturer's protocols. Protein concentrations were determined by Bradford method using bovine serum albumin as a standard.

### 2.14. RNA Isolation, RT-PCR, and Quantitative Real-Time PCR Analyses

Total RNA was isolated from liver tissues using TRIzol reagent (Takara Biotechnology, Dalian, China) and then reversely transcribed to cDNAs using PrimeScript RT Master Mix (Takara) according to the manufacturer's instructions. Quantitative real-time PCR analysis was performed using SYBR Green Master Mix (Takara) in a Roche LightCycler® 480 System (Roche Group, Switzerland). GAPDH was analyzed in each sample as an internal control for normalization, and fold changes in mRNA expression were calculated by the Comparative-Ct Method (ΔΔCt method).

Forward and reverse primers used for specific genes are listed as follows: Bax: (Forward, 5′-TTTCATCCAGGATCGAGCAGG-3′ and Reverse, 5′-GCAAAGTAGAAGAGGGCAACCAC-3′); Bcl2: (Forward, 5′-GGCATCTTCTCCTTCCAG-3′ and Reverse, 5′-CTACCCAGCCTCCGTTAT-3′); GAPDH: (Forward, 5′-TGCCGCCTGGAGAAACCT-3′ and Reverse, 5′-TGAAGTCGCAGGAGACAACC-3′).

### 2.15. Western Blot Analysis

Frozen liver tissues were homogenized in lysis buffer with glass homogenizers, and protein concentrations were then determined. Proteins were separated by sodium dodecyl sulfate-polyacrylamide gel electrophoresis (SDS-PAGE), transferred onto polyvinylidene difluoride membranes, and then identified by immunoblot analysis with appropriate primary antibodies at a dilution of 1 : 1000. Antibodies against cleaved caspase-3 (9664), caspase-3 (9665), cleaved caspase-9 (9509), caspase-9 (9508), Bax (2772), Bcl2 (3498), phospho-JNK (4668), JNK (9252), phospho-ERK1/2 (4370), ERK1/2 (4695), GAPDH (2118), Nrf2 (12721), HO-1 (70081), Tubulin (2148), and Lamin B (13435) were purchased from Cell Signaling Technology (Beverly, MA, USA); antibodies against NQO1 (11451–1-AP) and CYP2E1 (19937–1-AP) were purchased from Proteintech Group (Wuhan, China). Horseradish peroxidase-conjugated antibodies to rabbit IgG (7074) or to mouse IgG (7076) (1 : 5000 dilution for each) were purchased from Jackson ImmunoResearch Laboratories (West Grove, PA, USA). Protein bands were visualized using a SuperSignal West Pico Kit (Thermo Fisher Scientific Pierce, IL, USA) according to the manufacturer's instructions.

### 2.16. HPLC Analysis of Main Compounds of FMF

Chemical composition of FMF was determined using HPLC. The analysis was performed using an HC-C_18_ column (5 *μ*m, 150 mm × 4.6 mm id, Agilent Technologies, USA) on an Agilent 1260 series HPLC system equipped with a diode array detector, an autosampler, and an openLAB CDS ChemStation Workstation (Agilent Technologies). A gradient elution was performed by varying the proportion of solvent A (acetonitrile-methanol, 25 : 75, *v*/*v*) to solvent B (water, containing 0.1% formic acid), with a flow rate of 1.0 mL/min. The solvent gradient was as follows: 0–10 min from 5% to 25% A; 10–50 min from 25% to 28% A; 50–70 min from 28% to 28% A. The wavelength for UV detection was 360 nm, and the injection volume was 10 *μ*L. All separations were performed at 25°C. The lyophilized powder of FMF was dissolved in methanol at a concentration of 0.44 mg/mL. All samples and mobile phases were filtered through a 0.45 *μ*m membrane filter (Millipore) and then degassed in an ultrasonic bath prior to use. Four different flavonoids, including vitexin, isovitexin, isorhamnetin-3-O-*β*-D-glucoside, and narcissin were investigated. Identification of these compounds was performed by comparing retention times (*t*_R_) with those of commercial standards. The linear regression equation for each calibration curve was established by plotting the amount of each standard compound injected against the average peak area.

### 2.17. Statistical Analysis

All data were representative of at least three independent experiments. Results were shown as mean ± SD. Student's *t*-test was used for comparisons between two groups. Differences were considered significant at *p* < 0.05. Prism 6 software package (GraphPad Software Inc., USA) was employed for statistical tests and graphical presentation of data.

## 3. Results

### 3.1. Total Phenolic, Flavonoid Contents, and Antioxidant Activities of Different Extracts

**Figure 1:**
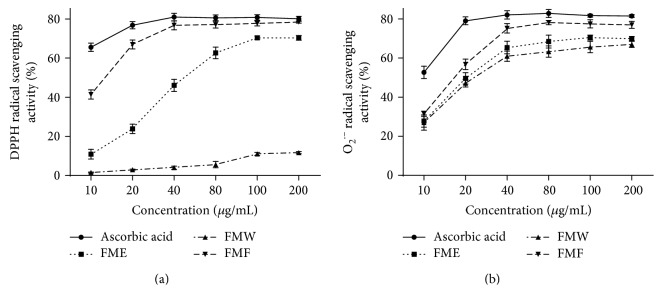
In vitro antioxidant activities of different extracts from *Folium Microcos*. (a) DPPH radical scavenging activity. (b) Superoxide anion radical (O_2_^•−^) scavenging activity. FME, the concentrated solution; FMW, the fraction eluted with water; FMF, the fraction eluted with 75% ethanol. Results are shown as mean ± SD (*n* = 3).

Polyphenolic compounds are able to alleviate oxidative stress by exerting antioxidant activities or affecting the antioxidant defense system [32, 33]. Therefore, total phenolic contents of FME, FMW, and FMF were determined using Folin-Ciocalteu method. As presented in Table 1, FME, FMW, and FMF contained 79.3 ± 1.1, 24.2 ± 2.5, and 338.1 ± 8.4 *μ*g GAE/mg extract, respectively. Similar results were observed from the determination of total flavonoid contents in FME, FMW, and FMF, with values reaching 175.8 ± 2.1, 36.0 ± 4.2, and 519.3 ± 5.3 *μ*g RE/mg extract, respectively. This quantitative analysis demonstrated that FMF contained the highest content of total phenolics and total flavonoids, implying that FMF is a polyphenol-enriched fraction. Then, DPPH free radical and superoxide anion radical (O_2_^•−^) scavenging systems were employed to evaluate the antioxidant potential of FME, FMW, and FMF. As shown in Figure 1 and Table S1 in Supplementary Material available online at https://doi.org/10.1155/2017/3631565, FMF exhibited a concentration-dependent scavenging activity against DPPH and O_2_^•−^ radicals ranging from 10 *μ*g/mL to 40 *μ*g/mL, and this property was superior to those of other tested extracts. In addition, the difference in free radical scavenging activity was unremarkable between FMF and the positive control ascorbic acid in both detected systems, especially when the concentration was above 40 *μ*g/mL. These results showed close connection between both total phenolic and flavonoid contents and antioxidant capacity, suggesting that polyphenol-enriched FMF from *Folium Microcos* may potentially provide protection against oxidative damage. Hence, FMF was selected for the following cell and animal studies.

### 3.2. Protective Effects of FMF on Oxidative Stress In Vitro

**Figure 2:**
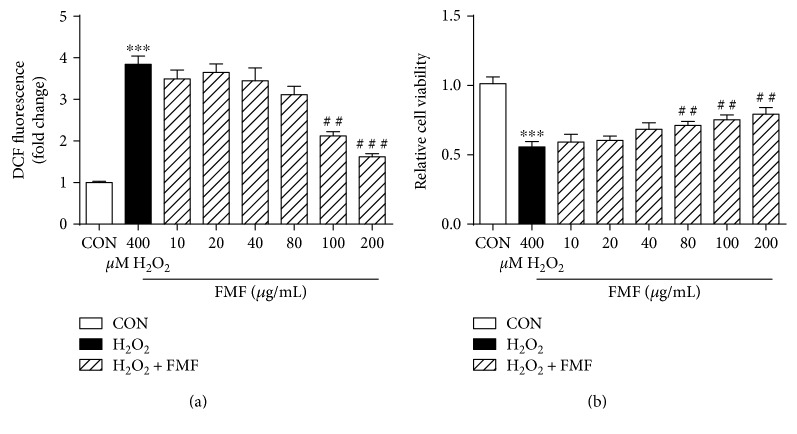
Effects of FMF on H_2_O_2_-mediated oxidative stress in HepG2 cells. Cells were treated with FMF (10, 20, 40, 80, 100, and 200 *μ*g/mL) in the presence of 400 *μ*M H_2_O_2_ for 4 h. (a) ROS formation was measured using a fluorescence microplate reader. (b) Cellular mortality was evaluated by MTT assay. Results are shown as mean ± SD (*n* = 3). ^∗∗∗^*P* < 0.001 compared with the control group; ^###^*P* < 0.001, ^##^*p* < 0.01 compared with H_2_O_2_-intoxicated group.

A H_2_O_2_-mediated oxidative stress model in HepG2 cells was established to investigate the effects of FMF on oxidative damage. As can be seen in Figure 2(), H_2_O_2_ exposure caused severe oxidative stress, as evidenced by increased ROS production and high cellular mortality compared with those by the untreated control cells. Prominent morphological changes were also caused by H_2_O_2_ stimulation (Figure S1). However, the abovementioned alterations were significantly alleviated by FMF pretreatment in a dose-dependent manner. Similar results were observed in Hepa1–6 cells (Figures S2 and S3). Further cytotoxicity assay indicated that FMF hardly affected the viability of tested cells (Figure S4). Collectively, these in vitro data supported the therapeutic potential of FMF in modulating antioxidant responses.

### 3.3. FMF Inhibited APAP-Induced Liver Injury

**Figure 3:**
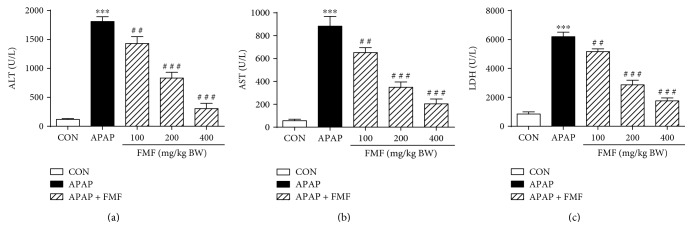
Effects of FMF on APAP-induced liver injury. Mice were intragastrically administered with either PBS or FMF at 100, 200, and 400 mg/kg body weight once daily for 7 consecutive days prior to single administration of APAP (500 mg/kg BW). Mice were killed at 12 h after APAP challenge. Activities of (a) ALT, (b) AST, and (c) LDH were measured in plasma samples by using the commercial kits. Results are shown as mean ± SD (*n* = 8 mice in each group). ^∗∗∗^*p* < 0.001 compared with the control group; ^###^*p* < 0.001, ^##^*p* < 0.01 compared with APAP-intoxicated group.

**Figure 4:**
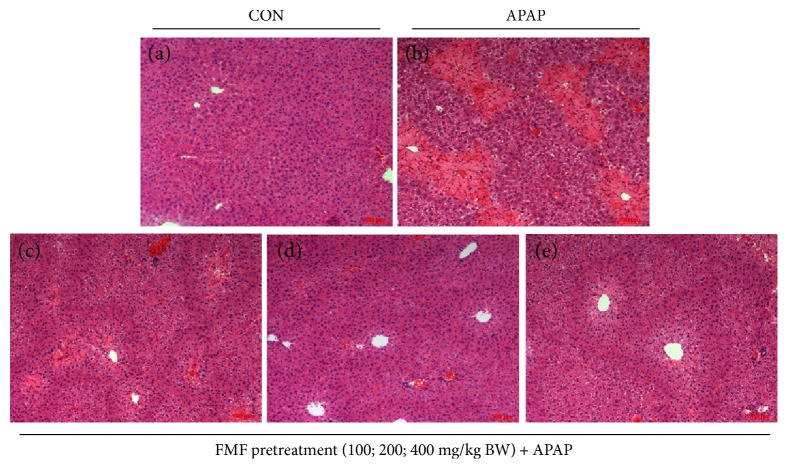
Effects of FMF on histological changes in APAP-intoxicated mice livers. Liver sections were stained with hematoxylin and eosin (original magnification of 100x). (a) Control group. (b) APAP-intoxicated group. (c) FMF (100 mg/kg BW) + APAP. (d) FMF (200 mg/kg BW) + APAP. (e) FMF (400 mg/kg BW) + APAP.

Oxidative stress plays an initial and augmented role in the pathogenesis of APAP-induced hepatotoxicity [34]. Therefore, hepatoprotective effects of FMF were investigated in APAP-induced liver injury mice model. As depicted in Figure 3, mice treated with APAP (500 mg/kg body weight) showed evidence of severe liver injury, as indicated by remarkable increases in serum ALT, AST, and LDH activities. However, these APAP-caused increases were significantly and dose-dependently inhibited by FMF pretreatment (100, 200, and 400 mg/kg body weight). Histopathological analysis provided supportive evidence for biochemical parameters assay (Figure 4). The sections of liver samples taken from APAP-intoxicated mice revealed extensive histological changes in the form of remarkable gross necrosis, sinusoidal congestion, hemorrhage, and inflammatory cell infiltration. Interestingly, FMF pretreatment remarkably ameliorated APAP-induced liver injury, and administration of APAP along with FMF at a dose of 400 mg/kg body weight showed a near-normal appearance, suggesting that FMF can protect against APAP-induced hepatic damage.

### 3.4. FMF Reduced APAP-Induced Hepatic Oxidative Stress

**Figure 5:**
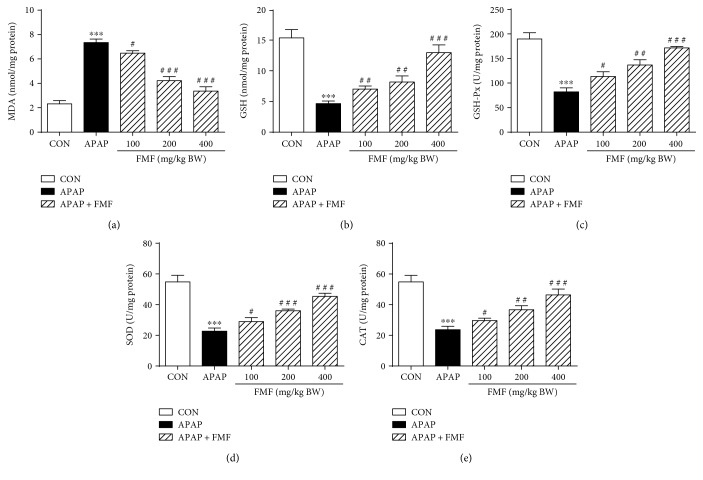
Effects of FMF on hepatic levels of MDA, GSH, GSH-Px, SOD, and CAT. Mice were intragastrically administered with either PBS or FMF at 100, 200, and 400 mg/kg body weight once daily for 7 consecutive days prior to single administration of APAP (500 mg/kg BW). Mice were killed at 12 h after APAP challenge. Liver samples were collected, and hepatic homogenates were used for the determination of (a) MDA, (b) GSH, (c) GSH-Px, (d) SOD, and (e) CAT levels by using the commercial kits. Results are shown as mean ± SD (*n* = 8 mice in each group). ^∗∗∗^*p* < 0.001 compared with the control group; ^###^*p* < 0.001, ^##^*p* < 0.01, ^#^*p* < 0.05 compared with APAP-intoxicated group.

Lipid peroxidation and antioxidant enzyme activities were measured to examine the role of FMF in APAP-induced oxidative damage. MDA, a major end product that forms during the final stages of lipid peroxidation, is generally recognized as a direct index of toxic processes caused by free radicals [35]. As shown in Figure 5, administration of APAP to mice remarkably elevated MDA levels as compared to the untreated normal group. The liver antioxidant capacity of mice was also sharply decreased, as manifested by the significant reduction in hepatic GSH, GSH-Px, SOD, and CAT activities, which are the major enzymatic or nonenzymatic antioxidants and regulators of tissues responsible for intracellular redox homeostasis. However, the abovementioned alterations were effectively disputed by FMF pretreatment, clearly indicating the strong antioxidant capacity of FMF against APAP-induced liver injury.

### 3.5. FMF Ameliorated the Activation of ROS/MAPKs/Apoptosis Signaling and Facilitated Nrf2 Nuclear Translocation to Protect against APAP-Induced Hepatotoxicity

**Figure 6:**
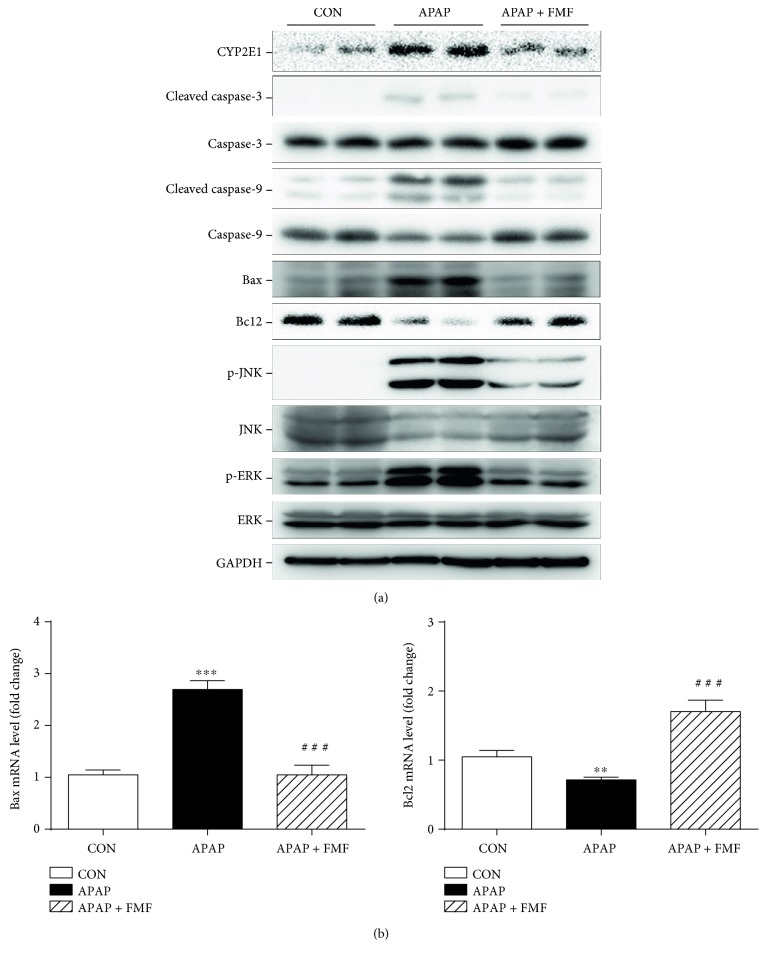
Effects of FMF on APAP-induced cell death-related gene expression. (a) Total cellular protein from liver tissues was extracted, and protein levels of CYP2E1, cleaved caspase-3/9, caspase-3/9, Bax, Bcl2, p-JNK, JNK, p-ERK, and ERK were determined by western blot. GAPDH was used as an endogenous control. (b) Total RNA from liver tissues was isolated and reverse-transcribed into cDNA for quantitative real-time PCR analysis of Bax and Bcl2 mRNA levels. GAPDH was used as an endogenous control. Results are shown as mean ± SD (*n* = 3). ^∗∗∗^*p* < 0.001, ^∗∗^*p* < 0.01 compared with the control group; ^###^*p* < 0.001 compared with APAP-intoxicated group.

**Figure 7:**
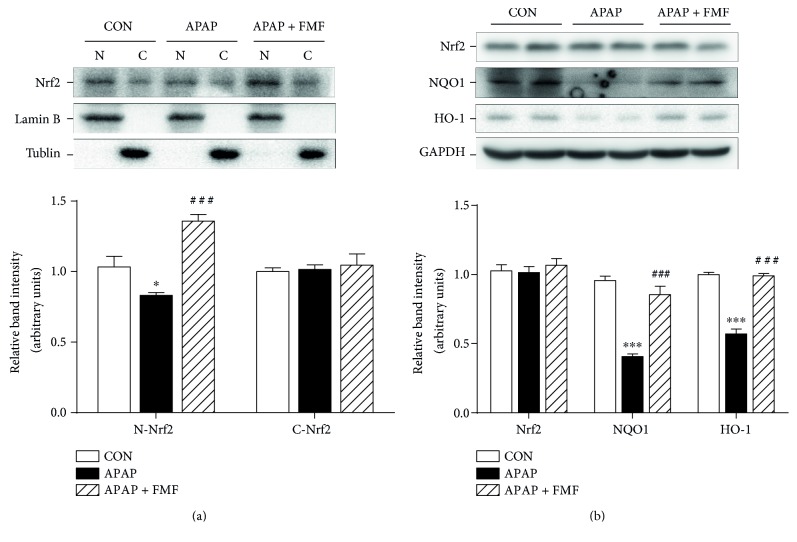
Effects of FMF on Nrf2 nuclear translocation and its target gene expression. (a) Nuclear and cytoplasmic extracts of liver tissues were prepared and protein level of Nrf2 was determined by western blot. Lamin B and tubulin were used as endogenous controls for nucleus and cytoplasm, respectively. (b) Total cellular protein from liver tissues was extracted, and protein levels of Nrf2, NQO1, and HO-1 were determined by western blot. GAPDH was used as an endogenous control. Relative intensity of the immunoreactive bands was analyzed and results are shown as mean ± SD (*n* = 3). ^∗∗∗^*p* < 0.001, ^∗^*p* < 0.05 compared with the control group; ^###^*p* < 0.001 compared with APAP-intoxicated group.

To determine the underlying mechanisms by which FMF alleviated APAP-induced acute liver injury, liver samples from mice treated with APAP with or without FMF pretreatment (400 mg/kg body weight) were homogenized for further western blot analysis. It is commonly recognized that APAP is converted to the highly reactive metabolite NAPQI by the hepatic cytochrome P450 system, especially CYP2E1, whose activity is critical for the development of APAP-induced hepatic injury [6, 7]. As presented in Figure 6(a), APAP treatment caused a significant increase in the expression level of CYP2E1. However, FMF pretreatment markedly suppressed APAP-induced elevation of CYP2E1. Many studies have illustrated the involvement of apoptosis in APAP-induced hepatotoxicity [36, 37]. Therefore, the expression of apoptosis-related proteins was determined. APAP administration significantly increased levels of cleaved caspase-3/9 and Bax and decreased Bcl2 protein expression. Nevertheless, these effects were prominently reversed by FMF pretreatment. Similar results were observed in mRNA levels of Bax and Bcl2 (Figure 6(b)). The activation of MAP kinases, especially the phosphorylation of JNK and ERK, can exacerbate APAP-induced mitochondrial oxidative stress and aggravate hepatic apoptosis [38]. As can be seen in Figure 6(a), administration of APAP to mice sharply increased levels of phosphorylated JNK and ERK. This was in good agreement with the results of previous studies. Interestingly, FMF pretreatment significantly inhibited APAP-mediated phosphorylation. Consequently, these results indicated that FMF alleviated APAP-induced hepatic apoptosis partially through inactivation of JNK/ERK MAPK pathway. Nrf2, which is a key sensor of oxidative stress, plays a protective role against APAP-induced hepatotoxicity by regulating the expression of intracellular detoxifying and antioxidant genes responsible for cytoprotective progress [12]. To examine whether FMF affects Nrf2 signaling, Nrf2 protein expression and nuclear accumulation were measured. As shown in Figure 7, despite a slight increase in Nrf2 protein level, the nuclear translocation of Nrf2 was suppressed after APAP intoxication. The hypothesis that APAP treatment repressed the transcriptional activity of Nrf2 was further supported by declined expression levels of two Nrf2 target genes (NQO1 and HO-1). However, the suppression caused by APAP administration was dramatically restored by FMF pretreatment. Furthermore, FMF treatment may to some extent promote Nrf2 nuclear translocation and its target gene expression, as verified by experiments in Hepa1–6 cells (Figure S5). Taken together, these data suggested that administration of FMF augmented Nrf2 nuclear accumulation, resulting in an enhanced antioxidant defense system to protect against APAP-induced oxidative injury.

### 3.6. Characterization of Chemical Composition of FMF

HPLC analysis was conducted to identify and quantify the major polyphenolic compounds present in FMF. HPLC chromatograms of standards and samples were shown in Figure S6. Under proposed HPLC analytical conditions, a good baseline separation was obtained for all the analytes, and four flavonoid glycosides, including vitexin, isovitexin, isorhamnetin-3-O-*β*-D-glucoside, and narcissin, were identified by comparing their retention times (21.3, 24.6, 45.3, and 47.2 min, resp.) with those of authentic standards. Quantitative data were calculated from their respective standard curves (Table S2). As shown in Figure S6 (b) and Table S2, narcissin (62.4 *μ*g/mg) was identified as the major phenolic compound in FMF, followed by isorhamnetin-3-O-*β*-D-glucoside (11.3 *μ*g/mg), isovitexin (10.6 *μ*g/mg), and vitexin (10.4 *μ*g/mg).

## 4. Discussion and Conclusions

In the present study, we demonstrate that the polyphenol-enriched fraction (FMF) from *Folium Microcos* has a preventive effect on APAP-induced hepatic apoptosis and oxidative injury. APAP-induced hepatotoxicity, as an experimentally convenient and clinically relevant animal model, is widely used to investigate liver injury associated with oxidative stress caused by ROS and other free radicals [39]. Previous studies on APAP toxicity have documented that oxidative stress is the major mechanism involved in the development of hepatotoxicity, and extensive defects of antioxidant defense systems were also observed in APAP-intoxicated tissues [34]. Currently, N-acetylcysteine (NAC), as a precursor of the antioxidant glutathione (GSH), serves as the primary and FDA-approved antidote for APAP poisoning. However, its efficacy is limited because of therapeutic window and adverse effects [40, 41]. Therefore, it is of great importance to develop effective therapeutic agents with little or no side effect.

Polyphenolic compounds, which are most abundantly present in green teas and herbal teas, have drawn increasing attention for their medicinal functions and health benefits, and the widely accepted mechanism behind these properties involves the reduction of oxidative stress by scavenging free radicals [42]. To our knowledge, no report links hepatoprotective effects with phenolic constituents of *Folium Microcos*. In this study, the polyphenol-enriched fraction (FMF) from *Folium Microcos* exerted a strong antioxidant property in DPPH and O_2_^•−^ scavenging assays (Figure 1) and prevented H_2_O_2_-induced ROS production and cell death (Figure 2). The considerable antioxidant effect of FMF, which is mainly attributed to the high content of polyphenol constituents, implies the potential of *Folium Microcos* in the prevention or attenuation of oxidative stress-mediated liver injury. This hypothesis was further verified by suppressing APAP-induced liver damage in mice pretreated with FMF. Our data showed significant increases in levels of serum ALT, AST, and LDH, which are considered as sensitive indicators of liver tissue damage, after APAP administration, as reported previously (Figure 3) [7, 10]. However, FMF pretreatment substantially prevented these elevations, suggesting that FMF not only preserved structural and functional integrity of hepatic cellular membrane but also protected liver tissues against toxic effects of APAP. Histopathological examination provided visual evidence for hepatoprotective effects of FMF, as manifested by the restoration toward histomorphological variations (Figure 4).

It is commonly recognized that enhanced lipid peroxidation and decreased functioning of enzymatic or nonenzymatic antioxidant defense systems are the major characteristics of APAP-induced hepatotoxicity [6]. In this study, administration of APAP to mice dramatically reduced liver antioxidant capacity, as characterized by remarkable increase in MDA content and decrease in hepatic levels of SOD, CAT, GSH-Px, and GSH, which are often regarded as indicators of oxidative stress response [6, 7, 10]. However, FMF pretreatment distinctly reversed the changes in parameters of hepatic oxidative damage (Figure 5). Collectively, together with histopathological evidence, the results of biochemical parameters assays in serum and liver tissue demonstrated that the protective effect of FMF on APAP-induced liver injury may result from the modification of endogenous antioxidant defense systems.

Accumulating evidence has demonstrated that hepatic apoptosis plays a central role in APAP-induced hepatotoxicity [36, 37]. In this process, ROS derived from APAP bioactivation directly activates JNK through MAPK pathway. Together with ROS, activated JNK can stimulate the expression of proapoptotic proteins and block the function of antiapoptotic proteins, leading to serious hepatotoxicity and cell apoptosis [38]. It was also reported that inhibition of JNK or hepatocyte-specific JNK knockout mice prevented the development of mitochondrial oxidative stress and decreased hepatic apoptosis and liver injury [43, 44]. Consistent with previous results, the present study showed that APAP administration caused severe impairment of antioxidant defense systems and activation of MAPK pathway. However, these alterations could be substantially reversed by FMF pretreatment (Figures 5 and 6), suggesting significant potential of FMF to protect against oxidative stress-induced hepatic apoptosis. Members of the caspase and Bcl2 families have been proposed to be crucial regulators of apoptotic response mediated by many agents. Several studies have also confirmed prominent apoptotic characteristics in APAP-intoxicated liver tissues, as evidenced by release of cytochrome c from the mitochondria, decreased Bcl2/Bax ratio, and increased activities of caspase-3, 6, 8, and 9 [45, 46]. In our hands, during APAP challenge, FMF pretreatment significantly disputed the conversion of caspase-3/9 into active forms, enhanced Bcl2 level, and reduced Bax expression in protein and mRNA levels, implying that actions of FMF against APAP-induced liver injury may be through the suppression of apoptosis (Figure 6). On this basis, our results indicated that in the presence of FMF, the decrease in ROS-mediated activation of MAPKs might act in conjunction with the reductive effect on apoptosis axis, resulting in attenuated oxidative stress and increased cell survival in response to APAP challenge.

Upon oxidative stress, the antioxidant defense system in organisms is frequently activated to provide protection against oxidative damage by maintaining cellular redox homeostasis [47]. Numerous studies have demonstrated the active involvement of Nrf2 in oxidative stress-mediated hepatotoxicity induced by APAP treatment [12–17]. To explore the possible mechanism of hepatoprotection, the present work investigated inductive effects of FMF on the activation of Nrf2 signaling. Our results revealed that FMF pretreatment reinforced Nrf2 nuclear accumulation and delayed its nuclear exclusion in response to APAP, resulting in enhanced transcriptional activities. The positive regulation of FMF on Nrf2 signaling was further manifested by the restoration of APAP-induced reduction of phase-2 enzymes, including NQO1 and HO-1 (Figure 7 and Figure S5), both of which are the major regulators that play critical roles in the elimination of ROS and toxic metabolites derived from the redox process in liver tissues. NQO1 enzymatically reduces NAPQI and prevents mitochondrial dysfunction caused by APAP [48]. HO-1 catalyzes the cleavage of heme to form biliverdin and diminishes intracellular ROS production [49]. Consequently, our results demonstrated that FMF enhanced hepatic defense system through the activation of Nrf2-mediated antioxidant and detoxifying gene expression to protect against APAP-induced liver injury.

In the current study, the main polyphenols present in FMF were identified and quantified by HPLC analysis to gain insights into the major active constituents responsible for its antioxidant and hepatoprotective effects. HPLC analysis clearly showed that narcissin (62.4 *μ*g/mg) was identified to be the prominent phenolic compound in FMF, followed by isorhamnetin-3-O-*β*-D-glucoside (11.3 *μ*g/mg), isovitexin (10.6 *μ*g/mg), and vitexin (10.4 *μ*g/mg) (Figure S6 and Table S2). Narcissin and isorhamnetin-3-O-*β*-D-glucoside share the same basic parent structure of isorhamnetin, which has been demonstrated to be not only an effective antioxidant agent but also a candidate drug for the prevention and treatment of liver diseases [50]. Isorhamnetin was reported to be efficacious in protecting hepatocytes against oxidative stress through the activation of AMPK pathways [51]. A recent study verified the hepatoprotective function of isorhamnetin in attenuating liver fibrosis by inhibiting TGF-*β*/Smad and Nrf2 signalings [52]. It was also shown that narcissin possessed significant antioxidant effects on non-enzyme-induced lipid peroxidation in isolated microsomes and increased hepatic cell viability in both CCl_4_- and t-BuOOH-induced injury models [53]. Isorhamnetin-3-O-*β*-D-glucopyranoside was described to alleviate the adverse effects of hepatic ethanol ingestion by enhancing the activities of alcohol-oxidizing enzymes, microsomal ethanol-oxidizing system, and aldehyde dehydrogenase [54]. Vitexin, isovitexin, and vitexin- and isovitexin-enriched extracts also showed antioxidant or hepatoprotective activities [55–57]. Therefore, the presence of narcissin, isorhamnetin-3-O-*β*-D-glucoside, vitexin, and isovitexin in FMF from *Folium Microcos* may be the main bioactive compounds contributing to its antioxidant and hepatoprotective properties. This assumption requires further verification and will be discussed in our future studies.

**Figure 8:**
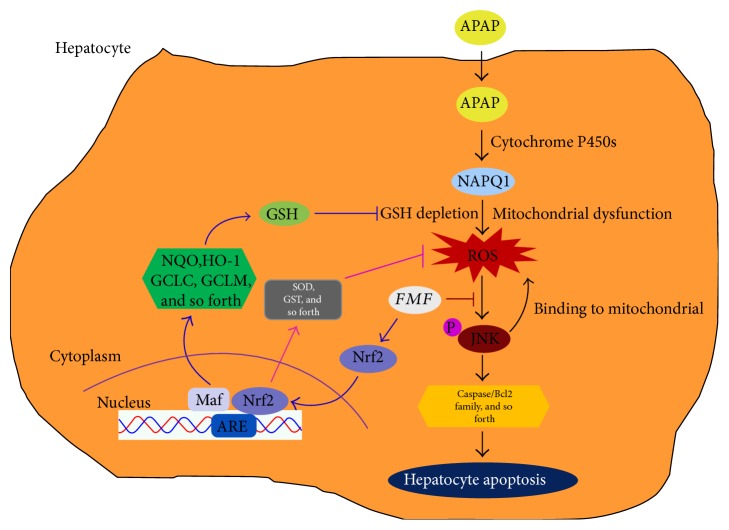
A proposed working model on how the polyphenol-enriched fraction (FMF) from *Folium Microcos* alleviates APAP-induced liver injury. After administration into the hepatocyte, APAP is first metabolized by cytochrome P4502E1 (CYP2E1) and generates NAPQI, which in turn depletes hepatic GSH and causes mitochondrial dysfunction, resulting in overproduction of ROS. ROS generated from mitochondria and other sources can cause the phosphorylation of JNK, which binds to the mitochondria, leading to enhanced ROS generation. Activated JNK can also phosphorylate transcription factors (e.g., c-Jun and NF-*κ*B) as well as members of the caspase and Bcl2 families to accelerate hepatocyte apoptosis, culminating in severe liver damage. Coadministration of FMF not only alleviated the changes in ROS/MAPKs-mediated apoptotic signaling cascade during APAP challenge but also promoted Nrf2 nuclear translocation and enhanced Nrf2-mediated antioxidant defense system, ultimately leading to reduced oxidative stress and cell death.

In conclusion, the current study demonstrates for the first time the hepatoprotective role of FMF in liver tissues as a critical crossroad in cascade reactions triggered by APAP challenge via dual modification of the apoptosis signaling through the effects on ROS/MAPKs axis and Nrf2-mediated antioxidant defense system response (Figure 8). Collectively, our data strongly revealed the clinical potential of the polyphenol-enriched fraction from *Folium Microcos* as a natural and functional food ingredient for the prevention of oxidative stress-induced hepatic injury.

## Supplementary Material

TABLE S1. In vitro antioxidant activities of different extracts from *Folium Microcos*. Figure S1: Effects of FMF on morphological changes induced by H_2_O_2_ in HepG2 cells. Cells were treated with FMF (10, 20, 40, 80, 100, and 200 μg/mL) in the presence of 400 μM H_2_O_2_ for 4 h and observed by microscope. (a) Control cells. (b) Cells exposed to H_2_O_2_. (c-h) Cells pretreated with different doses of FMF and then exposed to H_2_O_2_. Figure S2: Effects of FMF on H_2_O_2_-mediated oxidative stress in Hepa1-6 cells. Cells were treated with FMF (10, 20, 40, 80, 100, and 200 μg/mL) in the presence of 400 μM H_2_O_2_ for 4 h. (a) ROS formation was measured using a fluorescence microplate reader. (b) Cellular mortality was evaluated by MTT assay. Results are shown as mean ± SD (n=3). ^∗∗∗^ P < 0.001 compared with the control group; ^###^ p < 0.001, ^##^ p < 0.01, ^#^ p < 0.05 compared with H_2_O_2_-intoxicated group. Figure S3: Effects of FMF on morphological changes induced by H_2_O_2_ in Hepa1-6 cells. Cells were treated with FMF (10, 20, 40, 80, 100, and 200 μg/mL) in the presence of 400 μM H_2_O_2_ for 4 h and observed by microscope. (a) Control cells. (b) Cells exposed to H_2_O_2_. (c-h) Cells pretreated with different doses of FMF and then exposed to H_2_O_2_. Figure S4: Cytotoxicity assay. (a) HepG2 and (b) Hepa1-6 cells were treated with FMF (10, 20, 40, 80, 100, and 200 μg/mL) for 24 h, and cellular mortality was evaluated by MTT assay. Results are shown as mean ± SD (n=3). Figure S5: Effects of FMF on Nrf2 nuclear translocation and its target gene expression. Hepa1-6 cells were treated with FMF (100 μg/mL) for 12 and 24 h. (a) Nuclear and cytoplasmic extracts of cells were prepared, and the protein level of Nrf2 was determined by western blot. Lamin B and Tubulin were used as endogenous controls for nucleus and cytoplasm, respectively. (b)Total cellular protein was extracted, and protein levels of Nrf2, NQO1 and HO-1 were determined by western blot. GAPDH was used as an endogenous control. Relative intensity of the immunoreactive bands was analyzed, and results are shown as mean ± SD (n=3). ^∗∗∗^ p < 0.001, ^∗∗^ p < 0.01 compared with the control group. Figure S6: RP-HPLC profiles of (a) flavonoid standards and (b) flavonoid compounds in FMF at 360 nm. Peaks: 1, vitexin; 2, isovitexin; 3, isorhamnetin-3-O-β-D-glucoside; 4, narcissin.

## Figures and Tables

**Table 1:** Contents of total phenolics and total flavonoids of different *Folium Microcos* extracts.

Extract	Total phenolics (*μ*g GAE/mg extract)	Total flavonoids (*μ*g RE/mg extract)
FME	79.3 ± 1.1	175.8 ± 2.1
FMW	24.2 ± 2.5	36.0 ± 4.2
FMF	338.1 ± 8.4^a^	519.3 ± 5.3^a^

Results are shown as mean ± SD (*n* = 3). ^a^*p* < 0.001 compared with other extracts.
