# Supplementary material for: Hepatoprotective Effect of Polyphenol-Enriched Fraction from Folium Microcos on Oxidative Stress and Apoptosis in Acetaminophen-Induced Liver Injury in Mice
Source: Oxid Med Cell Longev. 2017 May 23;2017:3631565. doi: 10.1155/2017/3631565 (PMC5463195; doi:10.1155/2017/3631565)
Supplement: Supplementary file 1 — TABLE S1. In vitro antioxidant activities of different extracts from Folium Microcos. Figure S1: Effects of FMF on morphological changes induced by H2O2 in HepG2 cells. Cells were treated with FMF (10, 20, 40, 80, 100, and 200 μg/mL) in the presence of 400 μM H2O2 for 4 h and observed by microscope. (a) Control cells. (b) Cells exposed to H2O2. (c-h) Cells pretreated with different doses of FMF and then exposed to H2O2. Figure S2: Effects of FMF on H2O2-mediated oxidative stress in Hepa1-6 cells. Cells were treated with FMF (10, 20, 40, 80, 100, and 200 μg/mL) in the presence of 400 μM H2O2 for 4 h. (a) ROS formation was measured using a fluorescence microplate reader. (b) Cellular mortality was evaluated by MTT assay. Results are shown as mean ± SD (n=3). ∗∗∗ P < 0.001 compared with the control group; ### p < 0.001, ## p < 0.01, # p < 0.05 compared with H2O2-intoxicated group. Figure S3: Effects of FMF on morphological changes induced by H2O2 in Hepa1-6 cells. Cells were treated with FMF (10, 20, 40, 80, 100, and 200 μg/mL) in the presence of 400 μM H2O2 for 4 h and observed by microscope. (a) Control cells. (b) Cells exposed to H2O2. (c-h) Cells pretreated with different doses of FMF and then exposed to H2O2. Figure S4: Cytotoxicity assay. (a) HepG2 and (b) Hepa1-6 cells were treated with FMF (10, 20, 40, 80, 100, and 200 μg/mL) for 24 h, and cellular mortality was evaluated by MTT assay. Results are shown as mean ± SD (n=3). Figure S5: Effects of FMF on Nrf2 nuclear translocation and its target gene expression. Hepa1-6 cells were treated with FMF (100 μg/mL) for 12 and 24 h. (a) Nuclear and cytoplasmic extracts of cells were prepared, and the protein level of Nrf2 was determined by western blot. Lamin B and Tubulin were used as endogenous controls for nucleus and cytoplasm, respectively. (b)Total cellular protein was extracted, and protein levels of Nrf2, NQO1 and HO-1 were determined by western blot. GAPDH was used as an endogenous control. Relative intensity of [file 3631565.f1.pdf]

Supplemental Data

TABLE S1. In vitro antioxidant activities of different extracts from *Folium Microcos*.

Groups (μg/mg)	DPPH Radical Scavenging Activity (%)						$O_2^{\bullet-}$ Scavenging Activity (%)					
	10	20	40	80	100	120	10	20	40	80	100	120
**Vc**	65.55 ± 2.12	76.82 ± 1.85	80.97 ± 1.94	80.60 ± 1.46	80.76 ± 1.42	80.11 ± 1.22	52.69 ± 3.14	79.05 ± 2.05	82.06 ± 2.13	82.81 ± 2.02	81.68 ± 0.73	81.49 ± 0.74
**FMF**	$41.42 \pm 2.33^{***}$	$66.98 \pm 2.24^{**}$	76.79 ± 2.29^{ns}	77.08 ± 1.71^{ns}	77.74 ± 1.25^{ns}	78.44 ± 0.54^{ns}	$31.45 \pm 1.39^{***}$	$56.80 \pm 2.70^{***}$	$75.18 \pm 2.40^{*}$	$78.14 \pm 1.12^{*}$	$77.49 \pm 2.09^{*}$	$76.96 \pm 1.79^{*}$
**FME**	$10.87 \pm 2.46^{\#\#\#}$	$23.85 \pm 2.40^{\#\#\#}$	$46.06 \pm 3.09^{\#\#\#}$	$62.65 \pm 2.97^{\#\#}$	$70.37 \pm 0.94^{\#\#}$	$70.35 \pm 1.21^{\#\#}$	27.78 ± 3.07^{ns}	$49.63 \pm 2.90^{\#}$	$65.37 \pm 3.22^{\#}$	$68.43 \pm 3.31^{\#\#}$	$70.48 \pm 1.26^{\#\#}$	$69.95 \pm 1.04^{\#\#}$
**FMW**	$1.50 \pm 0.27^{\times\times\times}$	$2.94 \pm 0.29^{\times\times\times}$	$3.97 \pm 0.83^{\times\times\times}$	$5.57 \pm 1.61^{\times\times\times}$	$11.01 \pm 0.80^{\times\times\times}$	$11.66 \pm 0.57^{\times\times\times}$	27.02 ± 3.86^{ns}	$47.12 \pm 1.88^{\times\times}$	$60.97 \pm 2.63^{\times\times}$	$63.31 \pm 2.87^{\times\times}$	$65.60 \pm 2.90^{\times\times}$	$66.98 \pm 1.54^{\times\times}$

Results are shown as mean ± SD (n=3). ns, not significant, $^{***}p < 0.001$, $^{**}p < 0.01$, $^{*}p < 0.05$ compared with Vc group; $^{\#\#\#}p < 0.001$, $^{\#\#}p < 0.01$, $^{\#}p < 0.05$ compared with FMF group; $^{\times\times\times}p < 0.001$, $^{\times\times}p < 0.01$ compared with FMF group.

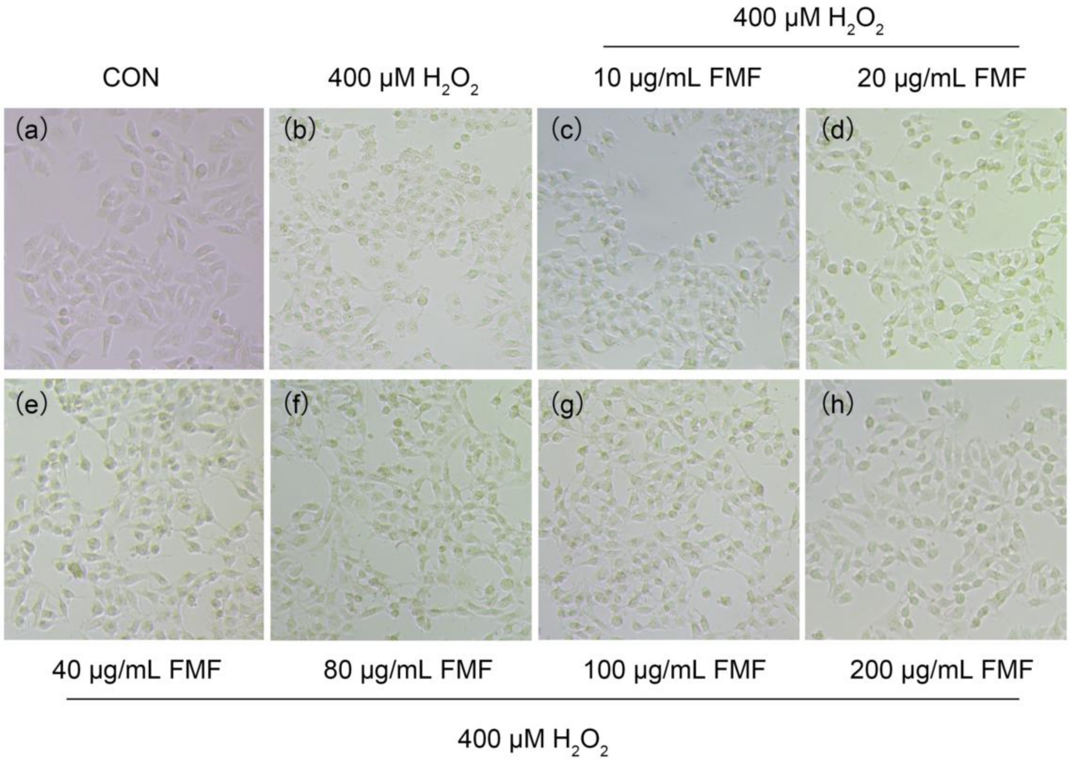

FIGURE S1: Effects of FMF on morphological changes induced by H_2O_2 in HepG2 cells. Cells were treated with FMF (10, 20, 40, 80, 100, and 200 μg/mL) in the presence of 400 μM H_2O_2 for 4 h and observed by microscope. (a) Control cells. (b) Cells exposed to H_2O_2. (c-h) Cells pretreated with different doses of FMF and then exposed to H_2O_2.

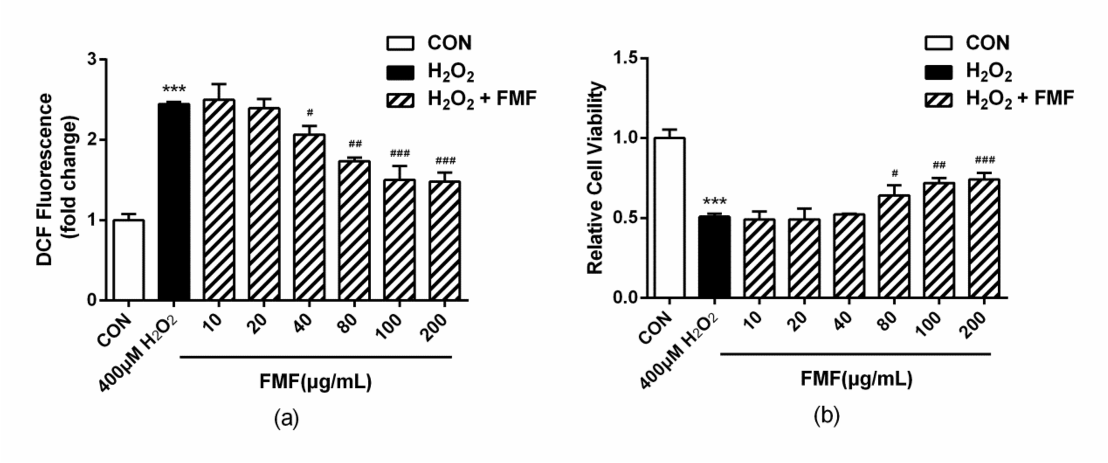

FIGURE S2: Effects of FMF on H_2O_2-mediated oxidative stress in Hepa1-6 cells. Cells were treated with FMF (10, 20, 40, 80, 100, and 200 μg/mL) in the presence of 400 μM H_2O_2 for 4 h. (a) ROS formation was measured using a fluorescence microplate reader. (b) Cellular mortality was evaluated by MTT assay. Results are shown as mean ± SD (n=3). *** $P < 0.001$ compared with the control group; $^{###}$ $p < 0.001$, $^{##}$ $p < 0.01$, $^{#}$ $p < 0.05$ compared with H_2O_2-intoxicated group.

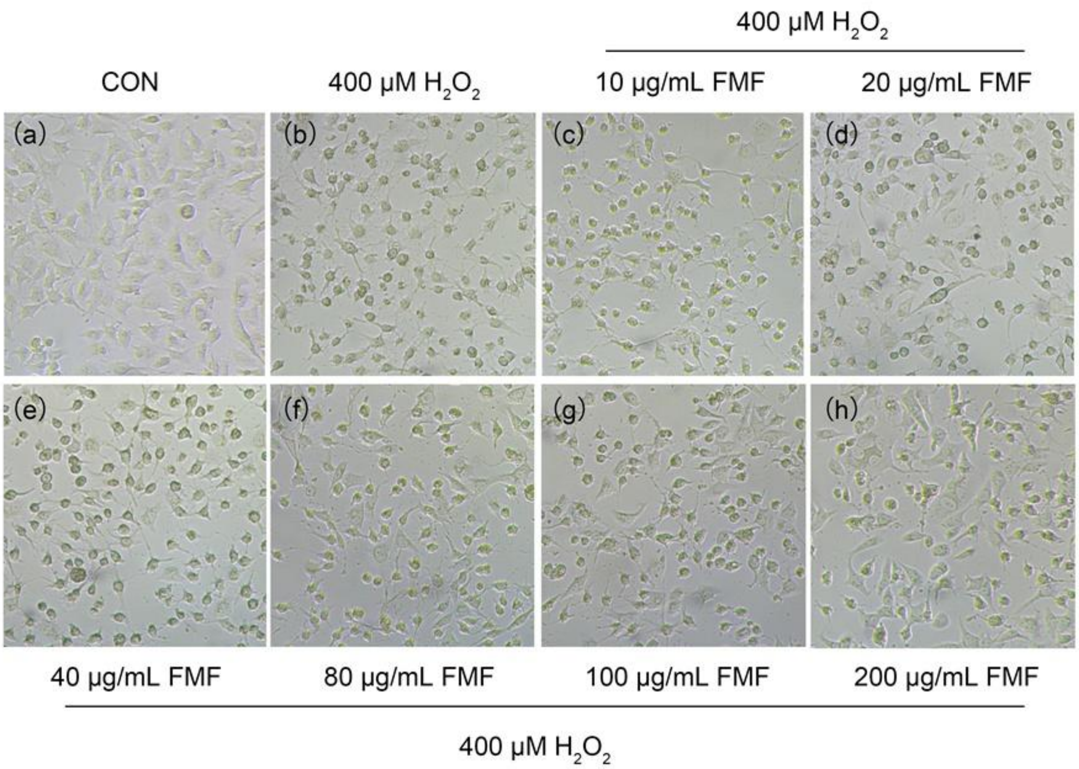

FIGURE S3: Effects of FMF on morphological changes induced by H_2O_2 in Hepa1-6 cells. Cells were treated with FMF (10, 20, 40, 80, 100, and 200 μg/mL) in the presence of 400 μM H_2O_2 for 4 h and observed by microscope. (a) Control cells. (b) Cells exposed to H_2O_2. (c-h) Cells

pretreated with different doses of FMF and then exposed to H_2O_2.

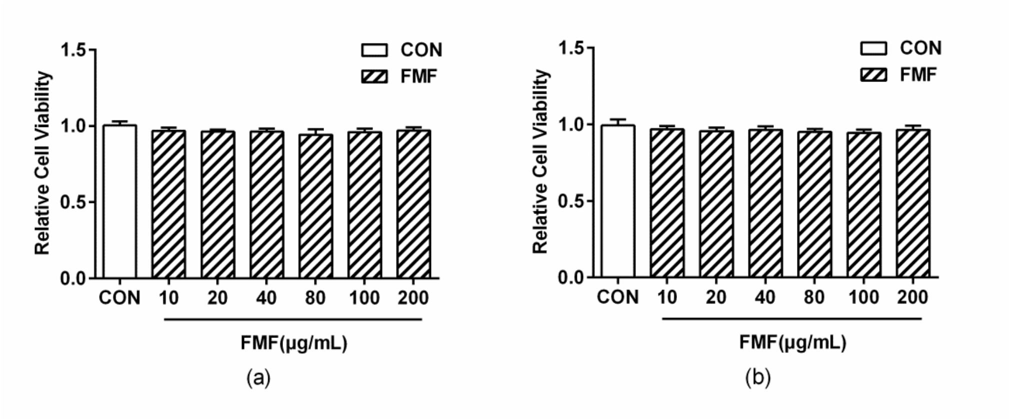

FIGURE S4: Cytotoxicity assay. (a) HepG2 and (b) Hepa1-6 cells were treated with FMF (10, 20, 40, 80, 100, and 200 μg/mL) for 24 h, and cellular mortality was evaluated by MTT assay. Results are shown as mean ± SD (n=3).

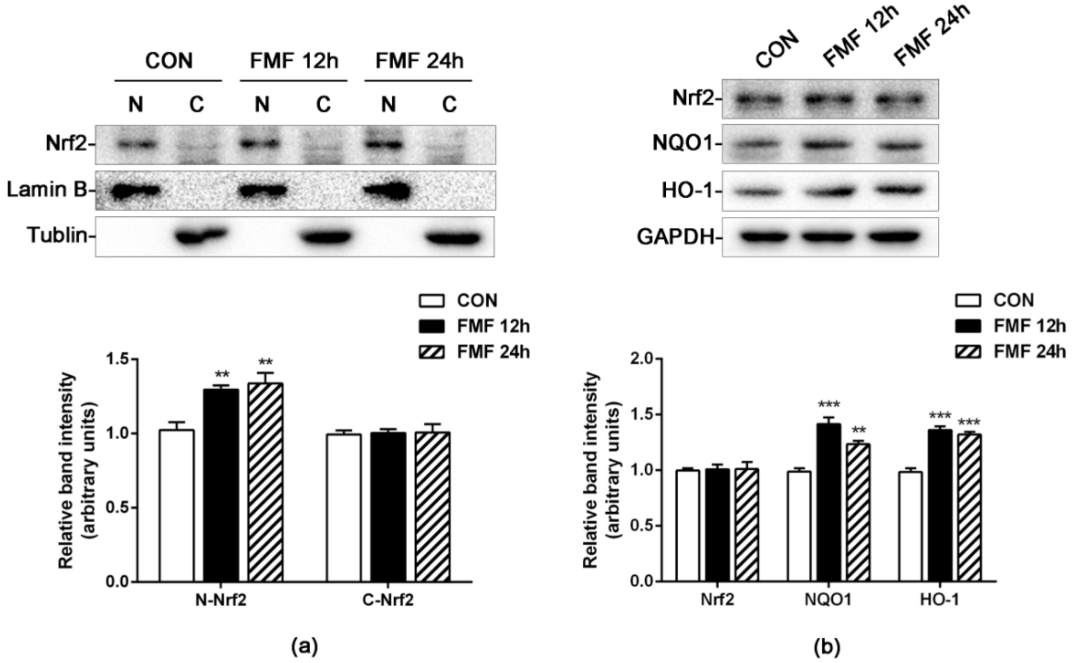

FIGURE S5: Effects of FMF on Nrf2 nuclear translocation and its target gene expression. Hepa1-6 cells were treated with FMF (100 μg/mL) for 12 and 24 h. (a) Nuclear and cytoplasmic extracts of cells were prepared, and the protein level of Nrf2 was determined by western blot. Lamin B and Tubulin were used as endogenous controls for nucleus and cytoplasm, respectively. (b)Total cellular protein was extracted, and protein levels of Nrf2, NQO1 and HO-1 were determined by western blot. GAPDH was used as an endogenous control. Relative intensity of the

immunoreactive bands was analyzed, and results are shown as mean ± SD (n=3). $^{***}p < 0.001$, $^{**}p < 0.01$ compared with the control group.

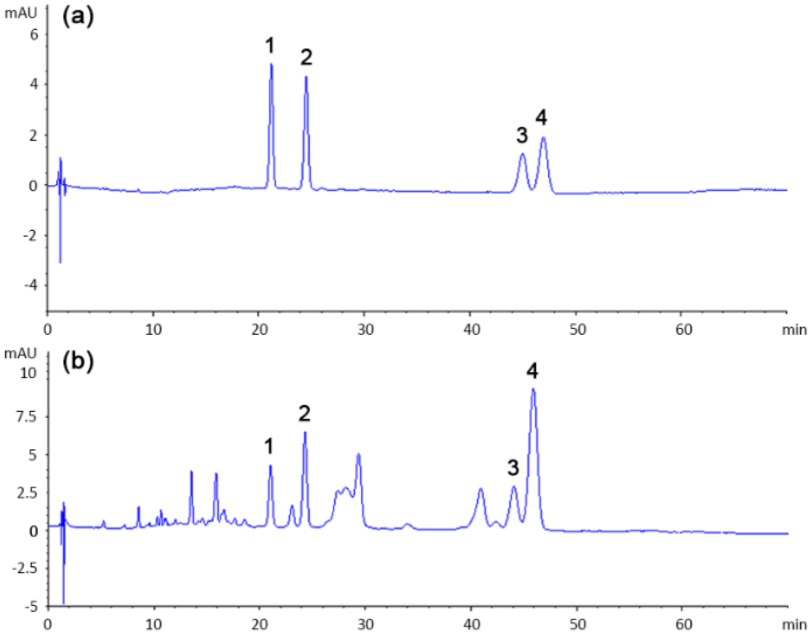

FIGURE S6: RP-HPLC profiles of (a) flavonoid standards and (b) flavonoid compounds in FMF at 360 nm. Peaks: 1, vitexin; 2, isovitexin; 3, isorhamnetin-3-O-β-D-glucoside; 4, narcissin.

TABLE S2: Calibration curves and contents of the polyphenolic compounds in FMF from *Folium Microcos*.

No	phenolic compound	content (μg/mg)	t_R (min)	equation of regression (Y = aX + b)	R^2
1	vitexin	10.37	21.27 ± 0.25	Y = 1744.0X + 16.472	0.9997
2	isovitexin	10.56	24.60 ± 0.21	Y = 3302.9X – 17.259	0.9995
3	isorhamnetin-3-O-β -D-glucoside	11.30	45.30 ± 0.26	Y = 3038.2X + 39.04	0.9994
4	narcissin	62.38	47.21 ± 0.32	Y = 1963.9X + 7.5613	0.9993
